# Seek first to understand

**DOI:** 10.1186/1747-5341-2-29

**Published:** 2007-11-28

**Authors:** Robert M Centor

**Affiliations:** 1 Division of General Internal Medicine, University of Alabama at Birmingham, Birmingham, AL 35294, USA; 2 Huntsville Regional Medical Campus, University of Alabama at Birmingham, Birmingham, AL 35294, USA

## Abstract

A recent study suggests that doctors often diminish effective time with patients by talking about themselves in a manner that does not improve the patient visit and is sometimes disruptive to it. Good care requires hearing what the patient has to say, as the doctor cannot set proper goals for a visit without knowing the patient's agenda. Listening to the patient is the key both to good patient care and to caring for the patient.

## Commentary

We spend much time in medical school learning cell biology, genetics, physiology, anatomy, pathophysiology, etc. Medical science dominates the curriculum. We must understand medical science to understand diagnosis and treatment. The science of medicine continues to progress, and our patients benefit greatly from this focus on scientific knowledge and the application of that knowledge.

But medicine is not solely a science. Great practitioners, while grounded in scientific principles, also excel in their interactions with patients. Patients often extol physicians who have great bedside manner. While we praise physicians with excellent bedside manner, we continue to have difficulty describing the features associated with excellent bedside manner despite a large and growing body of work on the subject [1-5].

A recent Archives of Internal Medicine article, "Physician self-disclosure in primary care visits enough about you, what about me? [6]" examined one aspect of the doctor patient relationship. While some may find their results surprising, I will argue that we should be able to predict and model good bedside manner.

This study of unannounced, undetected, standardized patients found that often physicians talk about themselves too much. Around one third of patient visits in that study included self-disclosure. These episodes of self-disclosure did not improve the doctor patient relationship, nor were these disclosures therapeutic. We should note that these patients were new to the physician, and thus the "self-disclosure" did not follow from a continuous interaction.

The study documents that physicians often talk about themselves rather than about the patient. They do not provide data on why physicians act this way. Rather they document the behavior, and find no apparent positive effect from this "self-disclosure." They suggest that the behavior can disrupt the physician patient relationship.

This study recalls the studies of physician interruption. Those classic studies document that physicians interrupt patient histories within 18 seconds (on average) [7,8]. Physicians apparently are quick to control the conversation.

Stephen Covey's classic book, "The 7 Habits of Highly Effective People [9]," includes the 5th Habit – Seek first to understand – then to be understood. I have read many discussions of this habit. How often do we emphasize this habit in the teaching of bedside manner?

The great physicians sit down, relax, and listen to the patient's story. They care (or least seem to care) about the patient's problems, and the context of those problems. I suspect they truly care, because listening is hard work.

If we do not understand the patient's concerns, then we cannot address those concerns. While we may discover other important problems to address, we cannot completely succeed unless we address the patient's original concerns.

Patients come to visits with their own agendas. We must respect those agendas and respond to those agendas.

Covey's words should influence each patient visit. We should take time to understand our patient. Once we have understood our patients, then we can actively address those concerns. If we have important things to explain to the patient, we should frame our concerns in the context of addressing the patient's agenda. Our agenda remains important, but I believe the patient's agenda should always influence how we present our agenda.

An anecdote may help make my points. Many years ago I cared for a 55 year old man with chronic heart failure. He remained functional, and continued to work. One day he came to see me and I immediately recognized that he had gained five pounds over the past month. My examination revealed increased work of breathing and bibasilar rales.

I spent five to ten minutes considering how to achieve outpatient diuresis. We developed a plan, and I planned to see him back in three days.

As I was about to leave the room, he asked me if I could help him with his shoulder. He told me that the reason for his visit was this shoulder pain. I examined him and asked about the shoulder pain. He told me that he had been taking over the counter non-steroidal anti-inflammatory drugs (NSAIDs). Now I understood his decreased diuresis.

If I had focused on his agenda first, I would have saved much time in addressing my agenda.

Patient visits should focus on the patient. If we believe that talking about ourselves will help the patient, then we can use a disclosure technique. We should think carefully about how a self-disclosure technique will help the patient.

The doctor patient visit is truly all about the patient. They come to our offices or hospitals so that we will address their concerns. We should always remember that context.

While we must inject our understanding of prevention and pathophysiology into the doctor patient interaction, our patients always deserve our concern about their concerns.

Originally said in 1925 by Francis Peabody, ". . . For the secret of the care of the patient is in caring for the patient [10]." The patient must occupy center stage in the doctor patient visit. Any activity that shifts that focus may harm the therapeutic relationship.

We must continue to advance the science of medicine. We must also continue to examine and teach the art of medicine. Science often helps patients, but artful medicine more often comforts patients.

I congratulate the authors of this study. Their work adds to a growing literature which studies bedside manner. We need more such studies. While we should never hope to script bedside manner, I suspect that more such studies will help us to teach and learn how to improve our own patient interactions.

## Competing interests

The author(s) declare that they have no competing interests.
